# How Helpful and What Is the Quality of Digital Sources of Healthy Lifestyle Information Used by Australian Adolescents? A Mixed Methods Study

**DOI:** 10.3390/ijerph182312844

**Published:** 2021-12-06

**Authors:** Matthew Armstrong, Nicole K. Halim, Rebecca Raeside, Si Si Jia, Karice Hyun, Farzaneh Boroumand, Mariam Mandoh, Anna C. Singleton, Philayrath Phongsavan, Julie Redfern, Stephanie R. Partridge

**Affiliations:** 1 Engagement and Co-Design Research Hub, Faculty of Medicine and Health, School of Health Sciences, The University of Sydney, Westmead 2145, Australia; marm9306@uni.sydney.edu.au (M.A.); nhal7837@uni.sydney.edu.au (N.K.H.); rebecca.raeside@sydney.edu.au (R.R.); sisi.jia@sydney.edu.au (S.S.J.); karice.hyun@sydney.edu.au (K.H.); farzaneh.boroumand@sydney.edu.au (F.B.); mariam.mandoh@sydney.edu.au (M.M.); anna.singleton@sydney.edu.au (A.C.S.); julie.redfern@sydney.edu.au (J.R.); 2 Prevention Research Collaboration, Faculty of Medicine and Health, Sydney School of Public Health, The University of Sydney, Sydney 2006, Australia; philayrath.phongsavan@sydney.edu.au; 3 ANZAC Research Institute, Concord Repatriation General Hospital, The University of Sydney, Sydney 2137, Australia; 4 Charles Perkins Centre, The University of Sydney, Sydney 2006, Australia; 5 The George Institute for Global Health, The University of New South Wales, Sydney 2006, Australia

**Keywords:** adolescents, social media, chronic disease, prevention, smartphone applications, websites, streaming services, nutrition, physical activity, obesity

## Abstract

To evaluate the digital platforms most used by adolescents for healthy lifestyle information, perceived helpfulness of platform information, helpfulness for positive behaviour changes, and quality of platforms’ lifestyle health information. Mixed-methods study including a cross-sectional online survey and content analysis. Eligible participants were 13–18-years; living in Australia; and had searched online for healthy lifestyle behaviour (nutrition, physical activity, weight management, sleep) information in the previous three months. Survey items examined the use of digital platforms, self-perceived helpfulness, usefulness for positive behaviour, and popular content. Data were analysed using descriptive statistics and ordinal logistic regression models. Content analysis was performed on popular digital content to evaluate expertise, objectivity, transparency, popularity, and relevance. In total, 297 participants completed the survey (62.3% female; 15.8 [SD1.5] years). Seventy-eight percent and 77% of participants reported using websites and social media, respectively, for seeking healthy lifestyle information. Websites and social media were rated as somewhat helpful by 43% and 46% of participants, respectively. Sixty-six percent and 53% of participants agreed/strongly agreed smartphone apps and social media were helpful for positive behaviour change, respectively. Helpfulness did not differ by age or gender. We evaluated 582 popular digital content; 38% were produced by a commercial company. Only 7% of content was from health organisations, 10% from health professionals and only 10% of content was objective, and 14% was transparent. Adolescents extensively utilise websites and social media for health information, yet popular content has limited objectivity and transparency. Governments and health organisations should consider creating age-appropriate digital information for healthy lifestyle behaviours.

## 1. Introduction

The global population of young people aged 10–24 years is now over 1.8 billion [1]. Adolescence is a period of rapid growth and development and is recognised as a critical life stage during which biological and socioenvironmental factors converge to engender lifestyle health-related trajectories into adulthood [2]. Few adolescents meet diet or physical activity guidelines globally [3,4], and adolescents in many countries do not get enough sleep on school days [5]. Research suggests that these behaviours are important predictors of adult health outcomes, such as obesity [6], and potentially may play a role in the pathophysiology of mental health disorders [7]. The global prevalence of overweight and obesity among children and adolescents has increased from 4% in 1975 to over 18% in 2016 [8]. In 2019, one in seven adolescents (approximately 175 million total) experienced a mental health condition, an estimated increase from four million in 2000 [9]. Consequently, adolescence presents an opportunistic window for implementing healthy lifestyle behaviours [2].

Digital platforms provide a range of opportunities for adolescents to engage with information and support to improve their lifestyle behaviours [10]. Education has been strongly linked to health outcomes and the use of preventive health services [11]. Moreover, digital technologies are accessible, with approximately 97% of people having mobile cellular service globally [12]. In Australia, 90% of adolescents own a mobile device or smartphone, and 95% access the internet daily [13]. As such, websites, and social media, are an increasingly popular approach for disseminating and sharing health information among this population [13]. International and national frameworks are also recognising that online health information influences adolescents to access health services and can improve adolescents’ engagement [10,14].

To date, most research has investigated the perceived barriers and facilitators to general health information-seeking behaviours among adolescents [15]. Studies have also sought to understand the broad methods and processes utilised by adolescents in their pursuit of health-related information [16]. However, there has been limited research regarding which digital platforms adolescents use for information about healthy lifestyle behaviours and the quality of information they seek. Additionally, rapid advancements and growth in the variety of digital platforms have outdated the current evidence-base. The uprise of contemporary social media platforms (e.g., TikTok) and on-demand video streaming services (e.g., Netflix) are yet to be analysed in their role of providing health information for adolescents. Therefore, the aim of this study is to evaluate the digital platforms most used by adolescents for healthy lifestyle information, perceived helpfulness of platform information, helpfulness for positive behaviour changes, and quality of platforms’ lifestyle health information.

## 2. Materials and Methods

### 2.1. Study Design and Participants

A mixed-methods study was undertaken, including a cross-sectional online survey, inclusive of qualitative and quantitative data, and a content analysis of popular digital content. The cross-sectional online survey was conducted between September and December 2020. Eligible participants were (i) 13–18 years of age (inclusive) which corresponds with secondary education in Australia; (ii) living in Australia; (iii) had searched for online health information at least once per month in the last three months using social media, smartphone apps, websites, or on-demand video/streaming ser-vices; and (iv) could read and understand English. A non-probability sampling method was used to recruit participants by paid social media advertising on Instagram and Facebook and through a market research company. Participants who completed the survey were offered the opportunity to enter a prize draw as an incentive for participation (one of 20, $50 gift vouchers). This study adhered to the Strengthening The Reporting of OBservational studies in Epidemiology (STROBE) statement checklist for reporting cross-sectional studies [17] (Supplementary Table S1). The study protocol was approved by the University of Sydney Human Research Ethics Committee (approval number 2020/613).

### 2.2. Survey Measures

An online survey was conducted using a secure survey platform. Participants were provided with a brief description of healthy lifestyle behaviours as all behaviours related to physical activity, weight management, diet or nutrition, and sleep. Participants were asked about: (A) demographic characteristics and lifestyle behaviours (B) use of the internet and electronic devices (C) use of preferred digital platforms for lifestyle information including (i) social media, (ii) smartphone apps, (iii) websites, (iv) on-demand video/streaming services, and (D) participants’ perceived helpfulness of digital platforms.

### 2.3. Demographic Characteristics and Healthy Lifestyle Behaviours

Specific questions on age, gender, participant educational status, parent or guardian education level, language spoken at home, and residential postcode (for categorizing socioeconomic Indexes for Areas (SEIFA) and major city, regional or remote residential areas) were derived from standardised Australian national surveys [18]. Participants’ residential postcodes were used to determine the SEIFA Index of Relative Socioeconomic Disadvantage (IRSD) quintile (quintile 5 representing the 20% most disadvantaged areas) [19] and the Australian Statistical Geographical Standard Remoteness Areas (ASGS-RA) as a measure of geographical location and relative access to services [20].

Participants were asked eight questions about healthy lifestyle behaviours. A validated single-item measure was used to assess physical activity frequency (days/week) [21]. Sleep duration (hours/day) was assessed using a single question adapted from the Pittsburgh Sleep Quality Index [22]. Self-reported height and weight was used to determine age-specific body mass index (BMI, kg/m^2^) [23]. Daily or weekly frequency of consuming specific foods and beverages (fruit, vegetables, energy-dense take-away meals, and sugar-sweetened beverages) were assessed using short validated dietary questions adapted from national population surveys [18]. Fruit consumption (serves/day) and vegetable consumption (serves/day) were assessed with a 7-point scale (0 serves to >5 serves). Sugar-sweetened beverage intake (cups/day) was assessed with a 6-point scale (0 cups to >4 cups). Participants were asked to report how frequently (each day, every week, every month, or never/rarely) they eat hot takeaway meals. Responses were converted to weekly takeaway consumption for analysis. Participants were also asked if they have any known chronic health conditions (yes [please specify], no, prefer not to answer) as defined by the National Strategic Framework for Chronic Conditions as encompassing a broad range of chronic and complex health conditions across the spectrum of illness, including mental illness, trauma, disability, and genetic disorders [24].

### 2.4. Use of Internet and Electronic Devices

Participants reported electronic device use (yes, no) and what types of devices (smartphone, wearable digital device, laptop or desktop computer or tablet device). Mobile internet data (gigabytes [GB]/month) was assessed with a 7-point scale (≤10 to >100). Participants were asked which digital platforms they used in the last 3 months for health information. Survey items, including social media, smartphone apps, websites, or on-demand video/streaming services. Platforms were selected based on current research trends of relevant, emerging, and influential online platforms within the current digital space.

### 2.5. Preferred Digital Platforms, Content, and Features

Participant opinions regarding preferred content or functional features of each digital platform to gain perspectives on optimising user engagement were captured with multiple-answer checkbox questions. Survey items for websites and smartphone apps were adapted from previous studies exploring the feasibility of these platforms for healthy lifestyle information-seeking purposes within the adolescent population [25,26,27]. Survey items for social media platforms and on-demand streaming services were developed by the research team due to limited existing survey questions exploring the functionality of such platforms.

### 2.6. Perceived Helpfulness of Digital Platforms for Seeking Healthy Lifestyle Information and Instigating Behaviour Change

Helpfulness of digital platforms for seeking healthy lifestyle information was assessed using a 5-point Likert scale (not at all helpful to extremely helpful). Likewise, the helpfulness of digital platforms for healthy lifestyle behaviour change was also assessed using a 5-point Likert scale (‘strongly disagree’ to ‘strongly agree’). Qualitative data via an open-ended question was captured to further understand how the four digital platforms have influenced the lifestyle behaviours of participants.

### 2.7. Popular Digital Content as Sources of Healthy Lifestyle Information

When participants reported using one or more of the digital platforms of interest, they were asked to list up to 3 of their favourite pages/persons/apps/shows/documentaries they follow, use, or view for regular healthy lifestyle updates from on (social media, websites, smartphone apps, and streaming services).

### 2.8. Data Analysis

A pilot survey found that approximately 75% of participants utilised websites to access healthy lifestyle information. Therefore, a sample size of 288 was estimated to capture a prevalence of 75%, with an absolute error of 5% and a type 1 error of 5%. Demographic characteristics were summarized using descriptive statistics. Continuous variables were summarised as means and standard deviations for normally distributed variables or as median and range for skewed variables. Categorical variables were summarised as numbers and percentages. Ordinal logistic regression models were used to explore the factors associated with participants finding helpfulness of accessing information and behaviour change for each digital platform. The factors were chosen according to statistical significance (*p* < 0.05) in the univariable analysis. SAS version 9.4 (SAS Institute Inc., Cary, NC, USA) was used for the quantitative analyses.

Each digital platform mentioned in the open-ended survey responses regarding perceived platform helpfulness underwent a content inductive analysis by one researcher (MA). An open coding approach was adopted to generate categories and subcategories as they emerged. This systematic approach is appropriate for open-ended survey questions to determine trends and patterns. A second researcher (SRP) familiar with the data was consulted to confirm the emerging themes from coding. Verbatim quotes that best represented the key findings for each category were used for the qualitative summary.

A content quality analysis was undertaken using all popular digital content reported by participants (top 3 favourite pages/persons/apps/shows/documentaries). A study-specific coding scheme was developed based on previous methodologies as there is no universally recognised method of assessment across multiple digital platforms [28]. The final coding scheme graded content based on published indicators used by consumers (expertise, objectivity, transparency, popularity, and relevance; See Supplementary Table S2 for category descriptions). The coding scheme was designed to be broad to provide an overview of the quality of the content. The data was entered and coded into a purpose-built database using Microsoft Excel (Version 16.46, Microsoft, Redmond, Washington, United States) containing a frequency of content. Duplicate content (one platform mentioned by numerous participants) was tallied and removed. Responses were considered ‘invalid’ if they were unable to be found on the associated social media platform. Two research team members, including one university-qualified health professional, independently coded the data (MA, SRP). Initially, a subsample (20% of content) was double-coded and underwent an in-depth team review to check coding consistency. Since there were few resulting discrepancies, each researcher independently coded the remaining content. Any discrepancies were discussed until consensus within the team was reached. Descriptive statistics were derived for all content and each digital platform.

## 3. Results

### 3.1. Study Flow and Participants

A total of 297 participants completed the survey and informed e-consent was considered in the analysis. The flow of participants, recruitment costs, and reach are presented in Supplementary Figure S1. Participants had a mean age of 15.9 years (SD 1.5, range 13–18 years), 62.3% of the sample was female (*n* = 185/297), 79.8% resided within major cities of Australia, and 42.8% were from the highest SEIFA quintile (Table 1). Approximately 47% of participants searched for health information online 1–2 times per month, 26% once a week, and 27% more than once per week.

Just over half (56%) of participants reported a healthy weight range (BMI 18.5–24.9 kg/m^2^) (Table 1), with 17% of participants categorised as having overweight and 8.4% with obesity. Over 90% of participants did not meet national recommendations for vegetables or physical activity. Chronic medical conditions were reported by 35% of participants. Approximately two-thirds reported having allergic rhinitis (33/103, 32.0%); 26.2% reported asthma (27/103), and 18.4% reported eczema (19/103).

### 3.2. Preferred Digital Platforms, Content, and Features

Approximately 93% of adolescents owned a smartphone, and 88% owned a laptop or desktop computer; 92% had a mobile data plan, with 66% with less than 30 GB (Table 1). Of the digital platforms used for healthy lifestyle information, 78% of participants reported using websites, 77% used social media, 31% used smartphone apps, and 29% streaming services (Table 1). Participants reported using multiple digital platforms to seek healthy lifestyle information; 35% used two digital platforms, 29% used three, and 29% used one. Twenty-two participants (7.4%) reported using all four platforms for healthy lifestyle information.

**Figure 1:**
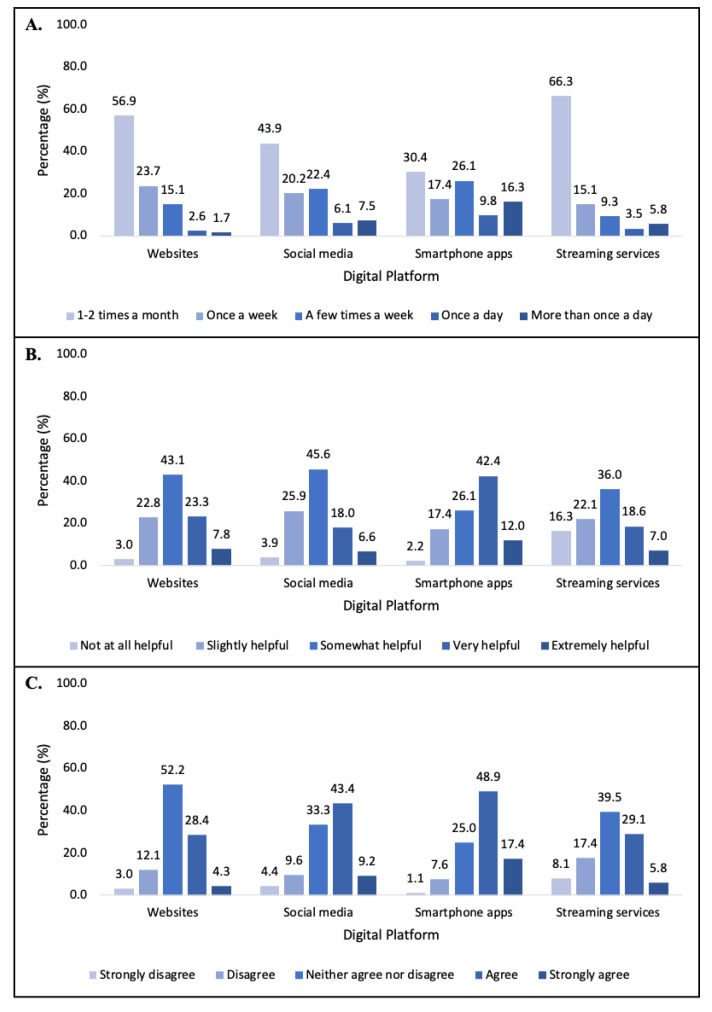
Survey items showing (**A**) Frequency of using digital platforms to access healthy lifestyle information; (**B**) Perceived helpfulness of digital platforms for accessing information and (**C**) Helpfulness of digital platforms for behaviour change.

Of participants who accessed healthy lifestyle information on websites, 57% reported a frequency of 1–2 times per week (Figure 1). Websites were reported as somewhat to extremely helpful by 74% of participants, and 52% neither agreed nor disagreed that websites were helpful for positive behaviour. Social media was used more frequently, with 56% of participants reporting use once per week or more frequently. Social media was considered somewhat helpful by 46% of participants and 43% agreed social media was helpful for positive behaviour change. Nearly 70% of participants reported using smartphone apps once per week or more frequently, with 54% considering apps very/extremely helpful, and 49% agreed/strongly agreed apps were helpful for positive behaviour change. Streaming services were used 1–2 times per month by 66% of participants to access healthy lifestyle information. Of those using the services, 36% reported streaming services as somewhat helpful for accessing healthy lifestyle information. Forty percent of participants neither agreed nor disagreed streaming services change healthy lifestyle behaviours.

Of website users, 81% preferred sites that were user-friendly, and 76% preferred sites containing information from credible or reputable sources (Supplementary Table S3). For participants using social media, 82% preferred browsing through content, and 60% preferred liking posts uploaded or shared by users, and for participants using smartphone apps, 84% preferred personalised content, and 80% preferred free apps. Of streaming service users, 67% preferred large content libraries, and 66% favoured low monthly membership fees.

Open-ended questions revealed components, categories, and attributes about the helpfulness of digital platforms for behaviour change (Table 2). The content inductive analysis found participants used websites to increase their general knowledge and be well-informed, find facts and increase awareness. In addition, websites were reported to positively change dietary behaviours by providing healthier recipes and increasing awareness of healthy dietary behaviours. Similarly, social media use was reported to have a positive impact on diet and physical activity behaviours by allowing the following of individual journeys and different types of people. Participants reported social media helped create a positive body image, improve their self-esteem and confidence. Smartphone apps were helpful for changing diet and physical activity behaviours through functional features such as tracking and accountability notifications. Streaming services influenced dietary behaviours by changing perceptions of the food industry and associated environmental impacts.

### 3.3. Perceived Helpfulness of Digital Platforms for Seeking Healthy Lifestyle Information and Instigating Positive Behaviour Change

Age and gender were not associated with the perceived helpfulness of social media, smartphone apps, websites, and streaming services for seeking information or for behaviour change (Supplementary Table S4). However, the data suggested that participants who consumed less than the recommended serving of fruit (<2 serves/day) found smartphone apps to be less helpful for seeking information than those who consumed the recommended servings of fruit (≥2 serves/day), adjusting for age and gender (Odd Ratio (95% Confidence Intervals): 9.86 (2.86, 33.93)).

### 3.4. Popular Digital Content as Sources of Healthy Lifestyle Information

Participants reported a total of 1318 pages, persons, apps, shows, or documentaries (hereafter referred to as ‘lifestyle content’) that they frequented for healthy lifestyle information, of which 771 (59%) were unique lifestyle content (i.e., after removing duplicate content) (Table 3). Of the unique sample, 582/771 (76%) lifestyle content was considered valid, which included 324 social media accounts, 153 websites, 71 smartphone apps, and 34 streams. Commercial companies and non-health professionals created 38% and 27% of the lifestyle content, respectively. Of the lifestyle content reported, 9.8% was considered objective, 14% was transparent, 63% related to physical activity, 57% to nutrition, 46% to weight management, and 16% to sleep. The most popular lifestyle content was reported by 30 participants (median 1, range 1–30). Only 5.7% of lifestyle content was repeatedly identified by more than five participants. Of lifestyle content reported, 22% and 1.2% of websites and social media accounts were by health organisations, respectively. The proportion of popular social media accounts by non-health professionals was 48%, and 17% were by health professionals. Popular social media accounts reported separately by more than five different participants had a median following of 2,958,000 (range 1,598,360–70,400,000). Case studies of the most popular content on each of the digital platforms are available in Supplementary Table S5.

## 4. Discussion

This study indicates that adolescents are primarily using websites and social media to seek healthy lifestyle information. Adolescents reported a diverse range of favourite content on digital platforms. Popular lifestyle content was from commercial companies or individuals, who were not health professionals with neither objective nor transparent content. Limited content was from credible sources such as health professionals or health organisations. There were no associations observed between demographic characteristics and the helpfulness of each digital platform. Overall, this study contributes new evidence on the perceived helpfulness and quality of digital platforms for healthy lifestyle purposes.

Almost half of the participants in this study used social media weekly for seeking healthy lifestyle information. This aligns with research that has identified social media as a significant source of health information among adolescents [29,30]. Previous studies suggest adolescents lack appropriate guidance for how they use social media for health purposes [31]. This is likely explained by the dynamic nature of social media platforms and the emergence of new features and platforms. Previous research has also established adolescents can appraise health information on websites confidently [29,32], however, it remains unknown how adolescents appraise health information on social media. Adolescents in this study reported a preference for browsing through content uploaded or shared by other users and reading through user-generated comments. Concerningly, our evaluation suggests the popular content reported by adolescents was generated by commercial companies or individuals who were not health professionals. Few participants reported content created by health organisations as their most favourite. As such, 72% of the popular content was influenced by commercial products or personal feelings and was rarely objective or transparent. In addition, social media has been reportedly linked to increased health risks, including poor weight perception, disordered eating, increased sedentary behaviours, and increased mental health concerns in young people [33]. Qualitative results from this study suggest social media also have the potential to create a positive body image and improve self-esteem amongst adolescents. These conflicting findings warrant that greater research impetus is placed in understanding the nature and influences of social media content and how it may present both benefits and risks to adolescents.

Websites and social media were perceived as helpful by adolescents for seeking health information and for behaviour change. Adolescents are more conducive to interactive and engaging health communication, and by design, social media is built for this purpose [26]. The qualitative survey data from this study support this position. Participants reported that they enjoy following other people’s lives and health journeys, which in turn motivate them to make positive changes to their diet or physical activity behaviours. Furthermore, the findings support previous research which has associated social media with positive behaviour change when utilised appropriately [34]. The benefits of social media are more apparent when compared to websites. Adolescents prefer interacting with visual tools, respond well to videos and personalised health experiences, and do not respond well to large bodies of text [26]. A recent study has indicated very few web pages were written specifically for adolescents, and no web pages were of excellent quality, highly interactive, and written in plain English [25]. Notably, in the present study adolescents were more likely to access websites that were from government and health organisations in comparison to other platforms.

The use of smartphone apps and streaming services for health information was reported by one-third of participants. Smartphone apps were reported by over 50% of participants as very helpful/extremely helpful for changing lifestyle behaviours, which further supports discourse on the positive impacts on lifestyle behaviours [27,35,36]. However, the infrequent uptake of this platform when compared to alternative digital platforms potentially warrants further investigation. Appropriately designed smartphone apps have been shown to improve physical activity and weight management behaviours, particularly through exercise trackers, fitness challenges, and goal setting [27]. Unlike smartphone apps, streaming services have yet to be formally analysed for their use for health purposes among adolescents. Previous research suggested significant behaviour change can result through popular streaming programs, such as ‘What the Health’, which was the most popular streaming content reported by participants in the present study. Such programs have been criticised for having distinctive viewpoints, misreporting evidence, and cherry-picking studies [37,38]. Given the compelling way healthy lifestyle content can be presented and the global reach and exposure they have, the implications of this platform warrant further attention.

There are several strengths and limitations in the study. A strength is that it provides insights into previously unevaluated uses of digital platforms for healthy lifestyle information among adolescents. Another strength of this study is the quality assessment of 582 content reported as ‘most popular’ across social media, smartphone apps, internet websites, and streaming services. Such an evaluation has not been undertaken and reveals what content adolescents find engaging. In the absence of universally recognised methods to examine the content quality of social media, websites, smartphones apps, and streaming services, a framework was adapted from previous methodologies [28]. Existing tools to examine the content quality of online information (e.g., DISCERN and Mobile App Rating Scale) could not be applied across multiple digital platforms. The more in-depth methodology is recommended for future research to understand the contextual differences of popular content across different digital platforms.

Due to the cross-sectional nature of the study, no causal relationships between helpfulness and demographic or health behaviours were able to be inferred. There are no validated questionnaires to evaluate helpfulness or preferred features of the four different digital platforms of interest in this study, and study-specific questions were developed. The convenience sampling method to recruit participants resulted in a non-representative sample. The persistent challenge associated with engaging adolescents in research is well reported [39]. Participants were female-skewed and from higher socio-economic areas in major cities. However, rates of key risk factors for chronic diseases, including overweight and obesity, fruit and vegetable intake, takeaway food intake, sleep, and physical activity levels were similar to national survey data [18]. Recruiting a larger and more representative sample of adolescents living in Australia remains a high research priority. A further limitation of this study is that it did not capture the digital literacy of participants. Previous research has shown adolescents can confidently seek health information online, however, they may not have the digital literacy skills to critically appraise this information [16]. Current digital literacy scales are in the content of internet-based health resources and do not factor in contemporary digital resources such as social media and streaming services [40]. Further research is recommended to assess the digital health literacy of adolescents in the context of healthy lifestyle information and across multiple contemporary digital platforms.

## 5. Conclusions

In conclusion, adolescents frequently used internet websites and social media for seeking healthy lifestyle information, yet popular content has limited objectivity and transparency. Given the rising rates of chronic disease risk factors among adolescents, this study has provided adolescents’ perspectives of emerging digital platforms and identified potential features and content to harness for health promotion. Governments and health organisations should consider creating age-appropriate digital information for healthy lifestyle behaviours. Future research should investigate whether the helpfulness of digital platforms is related to digital health literacy.

## Figures and Tables

**Table 1:** Demographic characteristics, healthy lifestyle behaviours and internet access, electronic devices, and digital platforms of participants (*n* = 297).

Demographic Characteristics		*n*	%
Age group (years)	13–14	58	19.5
	15–16	110	37.0
	17–18	129	43.4
Search frequency for lifestyle health information online	1–2 times a month	140	47.1
	Once a week	78	26.3
	A few times a week	56	18.9
	Once a day	10	3.4
	More than once a day	13	4.4
Gender identity	Male	105	35.4
	Female	185	62.3
	Other or prefer not to answer	7	2.4
Geographical location ^1^	Major city	237	79.8
	Regional or remote	56	18.9
	Other ^2^ or prefer not to answer	4	1.3
SEIFA quintile ^3^	Low (quintiles 1 and 2)	52	17.5
	Moderate (quintiles 3 and 4)	113	38.0
	High (quintile 5)	127	42.8
	Other ^2^ or prefer not to answer	5	1.7
Language other than English spoken at home	No	224	75.4
	Yes	73	24.6
Parent(s)/guardian(s) education level	Some high school	49	8.2
	Completed high school	67	11.3
	Studying for degree or diploma	19	3.2
	Trade or technical qualification	51	8.6
	Completed degree or diploma	230	38.7
	Post-graduate qualification	143	24.1
	Other, unknown, prefer not to answer	35	5.8
Currently attending high school	No	44	14.8
	Yes	253	85.2
Current work or education situation	Working casual, part-time, or full-time	23	7.7
	Enrolled in tertiary education course	27	9.3
Age-specific BMI categories ^4^	Underweight (BMI <18.5 kg/m^2^)	47	15.8
	Healthy (BMI 18.5–24.9 kg/m^2^)	165	55.6
	Overweight (BMI 25.0–29.9 kg/m^2^)	51	17.2
	Obese (BMI ≥30.0 kg/m^2^)	25	8.4
	Prefer not to answer	9	3.0
Chronic medical condition	No	190	64.0
	Yes	103	34.7
	Prefer not to answer	4	1.3
Vegetables (serves/day) ^5^	<5	269	90.6
	≥5	28	9.4
Fruits (serves/day) ^5^	<2	102	34.3
	≥2	195	65.7
Sugar sweetened beverages (cups/week)	0	189	63.6
	1–2	89	30.0
	≥3	19	6.4
Takeaway meals (meals/week)	Never/rarely	41	13.8
	Less than 1	106	35.7
	1–2	120	40.4
	3–4	25	8.4
	More than 4	5	1.7
Sleep (hours/day) ^5^	<8	228	76.8
	≥8	69	23.2
≥60-min of moderate or vigorous physical activity (days/week) ^5^	<7	270	90.9
	7	27	9.1
Wi-Fi access at home	Yes	297	100
	No	0	0.0
Electronic device ownership	Smartphone	277	93.3
	Wearable digital device	100	33.7
	Laptop or desktop computer	261	87.9
	Tablet device	112	37.7
Mobile data plan (GB/month) ^6^	0 GB	23	8.4
	≤30 GB	180	65.9
	>30 GB	48	17.6
	Don’t know	22	8.1
Online platforms utilised for health-related purposes	Internet websites	232	78.1
	Social media	228	76.8
	Smartphone apps	92	31.0
	Streaming services	86	29.0

^1^ Classified participant residential areas into five classes using postcode (major cities, inner regional, outer regional, remote, and very remote) were aggregated into two categories, major cities and regional/remote (inner regional, outer regional, remote, and very remote); ^2^ Postcode not listed to determine geographical location or SEIFA; ^3^ SEIFA provides measures of socioeconomic conditions by geographic area ranks areas in Australia according to relative socioeconomic disadvantage; ^4^ Determined using the defined ranges by the International Obesity Task Force; ^5^ Categories based on national recommendations for vegetables, fruit, physical activity, and sleep; ^6^ 24 participants had no data plan BMI, Body Mass Index; GB, gigabyte; SEIFA, Socioeconomic Indexes For Areas.

**Table 2:** Components, categories and attributes from the open-ended questions about helpfulness of digital platforms for behaviour change.

Digital Platform	Category	Attributes	Verbatim Quote (Gender, Age)
Social media (*n* = 120 responses)	Changed body image (*n* = 25)	Positive body image, improvements to self-esteem and confidence	“There is an emerging drive for positive stigma around different body shapes and sizes. This has helped me feel very comfortable with who I am.” Female, 17 years “There are lots of encouraging threads shared about positive body image, and it doesn’t matter if you have a bigger size than others, as long as you are happy & comfy.” Young person, 13 years
	2. Changed physical activity behaviours (*n* = 25)	Motivation, tips and ideas, free workouts	“Changed body workouts and structured the day into more organised and manageable loads.” Male, 15 years “I have become more active in the morning to do things. Get out of bed easier. Have more energy to do things. Wake up feeling good.” Female, 14 years
	3. Changed diet behaviours (*n* = 22)	Meal ideas, improved relationship with food	“I also love pages that post of quick healthy meal hacks.” Female, 17 years “I have also been influenced to view food as fuel and something that can be enjoyed in a balanced way, no need to complete restrict certain foods.” Female, 17 years
	4. Motivation by individuals (*n* = 19)	Following people’s journey’s, different types of people	“Seeing other people’s routines in regards to healthy eating and exercise has definitely influenced my lifestyle” Female, 18 years “[Understand] other people better, [seeing] people’s lifestyles and what food they eat in a day” Male, 13 years
Websites (*n* = 76 responses)	Changed general knowledge (*n* = 26)	Well informed, facts, awareness	“Information on these sites allow me to be more well informed on what actions to take in order to pay better attention to my heath” Male, 18 years “Helps gain knowledge on my health overall” Female, 16 years
	2. Changed diet behaviours (*n* = 22)	Healthier recipes, awareness of dietary behaviours	“More aware of what I’m consuming. Started cooking healthier meals because I had access to healthy recipes.” Female, 17 years
Smartphone apps (*n* = 23 responses)	Change physical activity behaviours (*n* = 23)	Wellbeing, tracking, accountability notifications	“Mainly keep track of my steps. I do at least 10 k steps a day and maybe 3x a week I hit 15 k. It means I spend more time outside and had helped my happiness and [wellbeing]” Female, 14 years “By providing exact speed and heart rate when I exercise, helping me control my routines.” Male, 13 years
	2. Changed diet behaviours (*n* = 15)	Notifications, tracking, accountability	“I can also access important information about sleep, nutrition and energy which helps me stick to a schedule.” Female, 18 years “The meal prep apps have given me a better understanding of what a well balanced meal should look like.” Female, 17 years
Streaming services (*n* = 30 responses)	Changed diet behaviours (*n* = 16)	Environmental impacts, vegetarian/vegan, less meat consumption	“Changed perception of meat and diary industry and how it is not very healthy” Male, 13 years “I eat less meat to help the environment” Female, 18 years

**Table 3:** Content analysis of popular pages, persons, apps, shows, or documentaries reported by participants.

Category	Sub-Category	All Content		Websites		Social Media		Smartphone Apps		Streaming Services	
		*n* = 582		*n* = 153		*n* = 324		*n* = 71		*n* = 34	
		*n*	%	*n*	%	*n*	%	*n*	%	*n*	%
Expertise	Individual health professional	59	10.1	4	2.6	55 *	17.0	0	0.0	0	0.0
	Individual non-health professional	158	27.1	4	2.6	154	47.5	0	0.0	0	0.0
	Health organisation	39	6.7	34	22.2	4	1.2	1	1.4	0	0.0
	Non-health organisation	4	0.7	0	0.0	4	1.2	0	0.0	0	0.0
	Commercial company	219	37.6	73	47.7	60	18.5	55	77.5	31	91.2
	Other	103	17.7	38	24.8	47	14.5	15	21.1	3	8.8
Objectivity	Commercial interests	420	72.2	81	52.9	254	78.4	55	77.5	30	88.2
	No commercial interests	57	9.8	32	20.9	23	7.1	1	1.4	1	2.9
	Cannot determine	105	18.0	40	26.1	47	14.5	15	21.1	3	8.8
Transparency	Disclosures	83	14.3	54	35.3	14	4.3	12	16.9	3	8.8
	Non-disclosures	397	68.2	59	38.6	263	81.2	44	62.0	31	91.2
	Cannot determine	105	18.0	40	26.1	47	14.5	15	21.1	3	8.8
Relevance	Nutrition	329	56.5	97	63.4	168	51.9	37	52.1	27	79.4
	Physical activity	369	63.4	113	73.9	169	52.2	56	78.9	31	91.2
	Weight management	268	46.0	86	56.2	126	38.9	40	56.3	16	47.1
	Sleep	92	15.8	54	35.3	20	6.2	15	21.1	3	8.8
	Cannot determine	64	11.0	40	26.1	6	1.9	15	21.1	3	8.8
Popularity	Content reported by ≥5 participants	33	5.7	9	5.9	14	4.3	8	11.3	2	5.9
		Median	Range	Median	Range	Median	Range	Median	Range	Median	Range
	Content frequency	1	1–30	1	1–30	1	1–30	1	1–16	1	1–16
	Number social media platforms for content with frequency ≥5	-	-	-	-	3	2–4	-	-	-	-
	Number of followers across platforms for content with frequency ≥5	-	-	-	-	2,958,000	1,598,360–70,400,000	-	-	-	-

* Inclusive of 35 accounts with expertise listed as ‘personal trainer’.

## Data Availability

The data presented in this study are available on request from the corresponding author. The data are not publicly available due to ethical and privacy restrictions.

## Supplementary Materials

The following are available online at https://www.mdpi.com/article/10.3390/ijerph182312844/s1, Table S1: STROBE Statement—Checklist of items that should be included in reports of cross-sectional studies. Table S2: Final coding scheme for content analysis. Table S3: Preferences for (i) digital platform and lifestyle behaviour (ii) content interaction or functional features by digital platform. Table S4: (A) Factors associated with helpfulness for accessing information for each digital platforms (B) Factors associated with helpfulness for behaviour change for each digital platforms. Table S5: Content analysis on the quality of most popular content from each digital platform. Figure S1: Flow of participants, recruitment costs and reach.

## References

[1] UNFPA (2014). The Power of 1.8 Billion—Adolescents, Youth, and the Transformation of the Future.

[2] Sawyer S.M., Azzopardi P.S., Wickremarathne D., Patton G.C. (2018). The age of adolescence. Lancet Child Adolesc. Health.

[3] van Sluijs E.M.F., Ekelund U., Crochemore-Silva I., Guthold R., Ha A., Lubans D., Oyeyemi A.L., Ding D., Katzmarzyk P.T. (2021). Physical activity behaviours in adolescence: Current evidence and opportunities for intervention. Lancet.

[4] UNICEF (2019). The State of the World’s Children 2019. Children, Food and Nutrition: Growing Well in a Changing World.

[5] Gariepy G., Danna S., Gobiņa I., Rasmussen M., Gaspar de Matos M., Tynjälä J., Janssen I.P.D., Kalman M.P.D., Villeruša A., Husarova D. (2020). How Are Adolescents Sleeping? Adolescent Sleep Patterns and Sociodemographic Differences in 24 European and North American Countries. J. Adolesc. Health.

[6] Mewton L., Champion K., Kay-Lambkin F., Sunderland M., Thornton L., Teesson M. (2019). Lifestyle risk indices in adolescence and their relationships to adolescent disease burden: Findings from an Australian national survey. BMC Public Health.

[7] Tolkien K., Bradburn S., Murgatroyd C. (2019). An anti-inflammatory diet as a potential intervention for depressive disorders: A systematic review and meta-analysis. Clin. Nutr..

[8] World Health Organisation Obesity and Overweight. https://www.who.int/news-room/fact-sheets/detail/obesity-and-overweight.

[9] Erskine H.E., Baxter A.J., Patton G., Moffitt T.E., Patel V., Whiteford H.A., Scott J.G. (2017). The global coverage of prevalence data for mental disorders in children and adolescents. Epidemiol. Psychiatr. Sci..

[10] World Health Organization (2020). Youth-Centred Digital Health Interventions: A Framework for Planning, Developing and Implementing Solutions with and for Young People.

[11] Albright A., Bundy D.A.P. (2018). The Global Partnership for Education: Forging a stronger partnership between health and education sectors to achieve the Sustainable Development Goals. Lancet Child Adolesc. Health.

[12] GSMA (2019). The State of Mobile Internet Connectivity 2019.

[13] eSafety Commissioner (2021). The Digital Lives of Aussie Teens.

[14] NSW Government (2017). NSW Youth Health Framework 2017–24.

[15] Park M.J., Scott J.T., Adams S.H., Brindis C.D., Irwin C.E. (2014). Adolescent and young adult health in the United States in the past decade: Little improvement and young adults remain worse off than adolescents. J. Adolesc. Health.

[16] Freeman J.L., Caldwell P.H.Y., Bennett P.A., Scott K.M. (2018). How Adolescents Search for and Appraise Online Health Information: A Systematic Review. J. Pediatr..

[17] von Elm E., Altman D.G., Egger M., Pocock S.J., Gøtzsche P.C., Vandenbroucke J.P. (2007). The Strengthening the Reporting of Observational Studies in Epidemiology (STROBE) statement: Guidelines for reporting observational studies. Ann. Intern. Med..

[18] Australian Bureau of Statistics (2015). 4364.0.55.001—National Health Survey: First Results, 2017–18.

[19] Australian Bureau of Statistics (2018). Socio-Economic Indexes for Areas (SEIFA): 2033.0.55.001.

[20] The Australian Bureau of Statistics The Australian Statistical Geography Standard: Remoteness Structure. https://www.abs.gov.au/websitedbs/d3310114.nsf/home/remoteness+structure.

[21] Scott J.J., Morgan P.J., Plotnikoff R.C., Lubans D.R. (2015). Reliability and validity of a single-item physical activity measure for adolescents. J. Paediatr. Child Health.

[22] de la Vega R., Tome-Pires C., Sole E., Racine M., Castarlenas E., Jensen M.P., Miro J. (2015). The Pittsburgh Sleep Quality Index: Validity and factor structure in young people. Psychol. Assess..

[23] Cole T.J., Lobstein T. (2012). Extended international (IOTF) body mass index cut-offs for thinness, overweight and obesity. Pediatr. Obes..

[24] Australian Health Ministers’ Advisory Council (2017). National Strategic Framework for Chronic Conditions.

[25] Ruan S., Raeside R., Singleton A., Redfern J., Partridge S.R. (2020). Limited engaging and interactive online health information for adolescents: A systematic review of Australian websites. Health Commun..

[26] Reen G.K., Muirhead L., Langdon D.W. (2019). Usability of health information websites designed for adolescents: Systematic review, neurodevelopmental model, and design brief. J. Med. Int. Res..

[27] Frontini R., Sousa P., Dixe M., Ferreira R., Figueiredo M. (2020). Designing a mobile app to promote healthy behaviors and prevent obesity: Analysis of adolescents’ preferences. Inform. Health Soc. Care.

[28] Sun Y., Zhang Y., Gwizdka J., Trace C.B. (2019). Consumer evaluation of the quality of online health information: Systematic literature review of relevant criteria and indicators. J. Med. Int. Res..

[29] Park E., Kwon M. (2018). Health-Related Internet Use by Children and Adolescents: Systematic Review. J. Med. Int. Res..

[30] Hausmann J.S., Touloumtzis C., White M.T., Colbert J.A., Gooding H.C. (2017). Adolescent and Young Adult Use of Social Media for Health and Its Implications. J. Adolesc. Health.

[31] Goodyear V.A., Armour K.M., Wood H. (2019). Young people and their engagement with health-related social media: New perspectives. Sport Educ. Soc..

[32] Freeman J.L., Caldwell P.H.Y., Scott K.M. (2020). The Role of Trust When Adolescents Search for and Appraise Online Health Information. J. Pediatr..

[33] Moorman E.L., Warnick J.L., Acharya R., Janicke D.M. (2020). The use of internet sources for nutritional information is linked to weight perception and disordered eating in young adolescents. Appetite.

[34] Chau M.M., Burgermaster M., Mamykina L. (2018). The use of social media in nutrition interventions for adolescents and young adults—A systematic review. Int. J. Med. Inform..

[35] Mollee J.S., Middelweerd A., Kurvers R.L., Klein M.C. (2017). What technological features are used in smartphone apps that promote physical activity? A review and content analysis. Pers. Ubiquitous Comput..

[36] Chan A., Kow R., Cheng J.K. (2017). Adolescents’ perceptions on smartphone applications (apps) for health management. J. Mob. Technol. Med..

[37] Pabian S., Hudders L., Poels K., Stoffelen F., De Backer C.J. (2020). Ninety Minutes to Reduce One’s Intention to Eat Meat: A Preliminary Experimental Investigation on the Effect of Watching the Cowspiracy Documentary on Intention to Reduce Meat Consumption. Front. Commun..

[38] Saell F. (2020). Female and male audiences’ perception on a plant-based (Vegan) diet after having viewed the documentary film What the Health: How perception on a plant-based diet (Vegan) changes after having watched the documentary film What the Health. Digit. Vetensk. Ark..

[39] Amon K.L., Campbell A.J., Hawke C., Steinbeck K. (2014). Facebook as a Recruitment Tool for Adolescent Health Research: A Systematic Review. Acad. Pediatr..

[40] Norman C.D., Skinner H.A. (2006). eHEALS: The eHealth Literacy Scale. J. Med. Int. Res..

