# Supplementary material for: How Helpful and What Is the Quality of Digital Sources of Healthy Lifestyle Information Used by Australian Adolescents? A Mixed Methods Study
Source: Int J Environ Res Public Health. 2021 Dec 6;18(23):12844. doi: 10.3390/ijerph182312844 (PMC8657837; doi:10.3390/ijerph182312844)
Supplement: Supplementary file 1 [file ijerph-18-12844-s001.zip › Digitalize Supplementary Table S2.pdf]

Supplementary Table S2. Final coding scheme[1] for content analysis

Category	Outcome measure
Expertise	Is the content creator an individual, organisation or company?
	Are there any details on health qualifications or health organisation?
Objectivity	Are there any commercial interests like products, programs or personal feelings?
Transparency	Are important information that influences a user's ability to make informed choices disclosed?
	Are commercial interests like products, programs clearly documented?
Popularity[2]	How many platforms does the content exist on (Instagram, Facebook, YouTube, TikTok)?
	What is the total number of followers on the platform (Instagram, Facebook, YouTube, TikTok)?
Relevance	Does the content have relevance to physical activity?
	Does the content have relevance to weight management?
	Does the content have relevance to nutrition?
	Does the content have relevance to sleep?

[1]Coding scheme developed based on previous methodologies reported. Social media accounts were coded utilising information available in the account name and biography, top 100 posts and captions accompanying content. Smartphone apps were coded using the Google play or iTunes stores description and app content. Websites were coded using the 'about us' section and content available on the page. Shows or documentaries available on streaming services were watched and reviewed. (18); [2]Popularity was only assessed for accounts with a reported frequency ≥ 5.
