# Supplementary material for: How Helpful and What Is the Quality of Digital Sources of Healthy Lifestyle Information Used by Australian Adolescents? A Mixed Methods Study
Source: Int J Environ Res Public Health. 2021 Dec 6;18(23):12844. doi: 10.3390/ijerph182312844 (PMC8657837; doi:10.3390/ijerph182312844)
Supplement: Supplementary file 1 [file ijerph-18-12844-s001.zip › Digitalize Supplementary Table S3.pdf]

Supplementary Table S3. Preferences for (i) digital platform and lifestyle behaviour (ii) content interaction or functional features by digital platform

Preference	Digital platform and lifestyle behaviour	n	%
Preferred platform to see more lifestyle health information (n=297)	Social media	149	50.2
	Smartphone apps	40	13.5
	Websites	79	26.6
	Streaming services	29	9.8
Preferred lifestyle behaviour to see more lifestyle health information (n=297)	Physical activity	109	36.7
	Nutrition	91	30.6
	Weight management	53	17.8
	Sleep	30	10.1
	Other	14	4.7
**Digital platform**	**Preferred content interaction or functional features**	**n**	**%**
Internet websites (n=232)	User-friendly	187	80.6
	Credible or reputable content	176	75.9
	Regularly updated information	149	64.2
	Age-appropriate information	149	64.2
	Visually appealing	120	51.7
	Social content or interactivity	56	24.1
	Other	2	0.9
Social media (n=228)	Browse through content	187	82.0
	Like posts uploaded or shared	136	59.6
	Reading through comments	114	50.0
	Reading status updates or captions	78	34.2
	Share content with other users	33	14.5
	Sign up or register for events	18	7.9
	Other(s)	2	0.9
Smartphone Apps (n=92)	Personalised or custom content	77	83.7
	Free	74	80.4
	Efficient or easy to follow	64	69.6
	Credible or reputable content	56	60.9
	Gamification features	38	41.3
	Social or interactive content	30	32.6
	Reminders or notifications	27	29.3
Streaming services (functional features) (n=86)	Large content library	58	67.4
	Low monthly or membership costs	57	66.3
	Enables offline downloads	45	52.3
	Higher quality video resolution	43	50.0
	Widespread device accessibility	41	47.7
	Free trial period	35	40.7
	Diversity of languages or captions	19	22.1
Streaming services (content features) (n=86)	Myth busters of 'hot topic' health trends and their impact on exercise performance, diet or weight loss	53	61.6
	Current trends and issues in food, health and pharmaceutical industries	52	60.5
	Impacts of lifestyle factors on environmental sustainability	50	58.1
	Documentaries following real life/personal experiences with a health journey	44	51.2
	Educational content on how to make lifestyle changes	36	41.9
	Food or culinary traditions and cultures	28	32.6
