# Supplementary material for: How Helpful and What Is the Quality of Digital Sources of Healthy Lifestyle Information Used by Australian Adolescents? A Mixed Methods Study
Source: Int J Environ Res Public Health. 2021 Dec 6;18(23):12844. doi: 10.3390/ijerph182312844 (PMC8657837; doi:10.3390/ijerph182312844)
Supplement: Supplementary file 1 [file ijerph-18-12844-s001.zip › Digitalize Supplementary Table S4.pdf]

Supplementary Table S4. A) Factors associated with helpfulness for accessing information for each digital platforms B) Factors associated with helpfulness for behaviour change for each digital platforms

**Characteristics, behaviours or health outcomes by helpfulness**		**Online platforms utilised for health-related purposes**							
		**Websites**		**Social media**		**Smartphone apps**		**Streaming services**	
		OR (95% CI)	*P*-value	OR (95% CI)	*P*-value	OR (95% CI)	*P*-value	OR (95% CI)	*P*-value
A. Helpfulness for accessing information									
Gender	Female vs Male	1.01 (0.62, 1.67)	0.9579	1.27 (0.76, 2.11)	0.3619	2.03 (0.6, 6.9)	0.2524	0.85 (0.36, 2.02)	0.7192
Age (years)	15 – 16 vs 13 – 14	0.64 (0.32, 1.3)	0.4179	1.03 (0.53, 2.03)	0.7545	3.08 (0.69, 13.78)	0.2862	1.31 (0.42, 4.16)	0.1762
	17 – 18 vs 13 –14	0.67 (0.34, 1.3)		1.23 (0.64, 2.36)		1.49 (0.31, 7.09)		0.57 (0.18, 1.78)	
Fruit (serves/day)[1]	< 2 vs ≥ 2	-	-	-	-	9.86 (2.86, 33.93)	<.0001	-	-
B. Helpfulness for behaviour change									
Gender	Female vs Male	0.84 (0.5, 1.41)	0.5106	0.88 (0.52, 1.49)	0.6231	2.29 (0.92, 5.71)	0.0749	0.86 (0.37, 2.02)	0.7311
Age	15 – 16 vs 13 – 14	1.06 (0.53, 2.11)	0.6968	0.83 (0.41, 1.66)	0.3961	1.74 (0.57, 5.34)	0.0976	0.75 (0.25, 2.28)	0.5558
	17 – 18 vs 13 –14	1.28 (0.66, 2.51)		1.24 (0.63, 2.44)		3.67 (1.08, 12.46)		0.55 (0.18, 1.67)	
Chronic medical condition	Yes vs No	-	-	-	-	0.7 (0.27, 1.85)	0.4719	-	-

[1] Australian national dietary guideline recommends two serves of fruit per day for adolescents, 13-18 years
