# Supplementary material for: How Helpful and What Is the Quality of Digital Sources of Healthy Lifestyle Information Used by Australian Adolescents? A Mixed Methods Study
Source: Int J Environ Res Public Health. 2021 Dec 6;18(23):12844. doi: 10.3390/ijerph182312844 (PMC8657837; doi:10.3390/ijerph182312844)
Supplement: Supplementary file 1 [file ijerph-18-12844-s001.zip › Digitalize Supplementary Table S5.pdf]

Supplementary Table S5. Content analysis on the quality of most popular content from each digital platform

Category	Digital platforms			
	**Social media**	**Smartphone apps**	**Websites**	**Streaming services**
**Name**	Chloe Ting	Fitbit	Healthline	What the Health
**Frequency**				
N, % of mentions by participants	30/568, 5.3%	16/156, 8.8%	30/314, 9.6%	16/64, 25.0%
**Expertise**				
Individual health professional<br>Individual non-health professional<br>Health organisation<br>Non-health organisation<br>Commercial company<br>Other	Individual health professional<br>• Personal trainer<br>• Other non-health qualifications listed	Commercial company<br>• Sell sensors and wireless technology	Commercial Company<br>• Health news website	Company<br>• Sensational documentary
**Objectivity**				
Are there any commercial interests like products or programs or personal feelings?	Yes<br>• Advertising present within posts e.g. Victoria's Secret advertising	Yes<br>• Sell sensors and wireless technology	Yes<br>• Advertise products and services e.g. Noom	Yes<br>• Selective interviewing by production team based on personal feelings
**Transparency**				
Are important information that influences a user's ability to make informed choices disclosed?	No<br>• Individual influencer<br>• No list of disclosures	Yes<br>• Terms of service listed on website	Yes<br>• Ads and sponsorship policy available	No<br>• Selective interviewing not disclosed by production team
**Popularity**				
Number of active platforms (Instagram, Facebook, YouTube, TikTok)	4	-	-	-
Number followers on Instagram, Facebook, YouTube, TikTok	24,018,234	-	-	-
**Relevance**				
Is there content relevant to physical activity?	Yes<br>• Workout programs	Yes<br>• App features to track physical activity	Yes<br>• Health topics include physical activity	No

**Category**	**Digital platforms**			
	**Social media**	**Smartphone apps**	**Websites**	**Streaming services**
Is there content relevant to weight management?	Yes<br>• Promoting a 'summer shred' challenge<br>• Body focused poses	Yes<br>• App features to track weight and weight loss	Yes<br>• Health topics weight management	Yes<br>• Content is relevant to weight management
Is there content relevant to nutrition?	Yes<br>• Recipe and meal ideas provided	Yes<br>• App features to track dietary intake	Yes<br>• Health topics include nutrition	Yes<br>• Content is relevant to nutrition
Is there content relevant to sleep?	No	Yes<br>• App features to track sleep	Yes<br>• Health topics include sleep	No
