# Supplementary figures and images for: How Helpful and What Is the Quality of Digital Sources of Healthy Lifestyle Information Used by Australian Adolescents? A Mixed Methods Study
Source: Int J Environ Res Public Health. 2021 Dec 6;18(23):12844. doi: 10.3390/ijerph182312844 (PMC8657837; doi:10.3390/ijerph182312844)

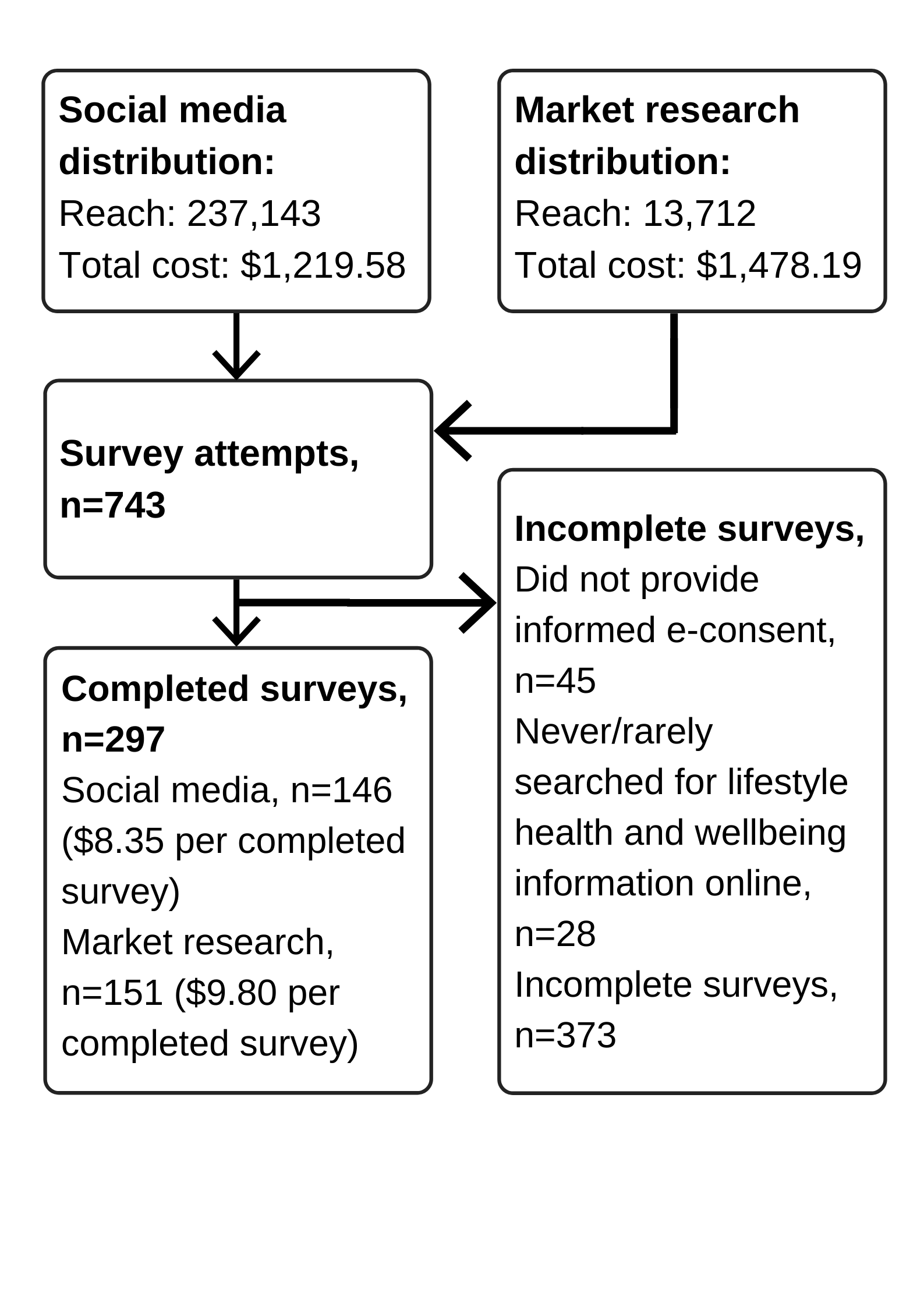

Supplement: Supplementary file 1 [file ijerph-18-12844-s001.zip › Digitalize Supplementary Figure S1.png]
